# Induction of an Inflammatory Loop by Interleukin-1β and Tumor Necrosis Factor-α Involves NF-kB and STAT-1 in Differentiated Human Neuroprogenitor Cells

**DOI:** 10.1371/journal.pone.0069585

**Published:** 2013-07-29

**Authors:** Subbiah Pugazhenthi, Yuji Zhang, Ron Bouchard, Gregory Mahaffey

**Affiliations:** 1 Section of Endocrinology, Veterans Affairs Medical Center, Denver, Colorado, United States of America; 2 Department of Medicine, University of Colorado Denver, Aurora, Colorado, United States of America; 3 Mayo Clinic, Rochester, Minnesota, United States of America; Oregon Health & Science University, United States of America

## Abstract

Proinflammatory cytokines secreted from microglia are known to induce a secondary immune response in astrocytes leading to an inflammatory loop. Cytokines also interfere with neurogenesis during aging and in neurodegenerative diseases. The present study examined the mechanism of induction of inflammatory mediators at the transcriptional level in human differentiated neuroprogenitor cells (NPCs). Interleukin-1β (IL-1β) and tumor necrosis factor-α (TNF-α) induced the expression of cytokines and chemokines in differentiated human NPCs as shown by an immune pathway-specific array. Network motif (NM) analysis of these genes revealed 118 three-node NMs, suggesting complex interactions between inflammatory mediators and transcription factors. Immunofluorescent staining showed increases in the levels of IL-8 and CXCL10 proteins in neurons and glial cells. Findings from Taqman low density array suggested the synergistic actions of IL-1β and TNF-α in the induction of a majority of inflammatory genes by a mechanism involving NF-kB and STAT-1. Nuclear localization of these transcription factors in differentiated NPCs was observed following exposure to IL-1α and TNF-α. Further studies on CXCL10, a chemokine known to be elevated in the Alzheimer's brain, showed that TNF-α is a stronger inducer of CXCL10 promoter when compared to IL-1β. The synergy between these cytokines was lost when ISRE or kB elements in CXCL10 promoter were mutated. Our findings suggest that the activation of inflammatory pathways in neurons and astrocytes through transcription factors including NF-kB and STAT-1 play important roles in neuroglial interactions and in sustaining the vicious cycle of inflammatory response.

## Introduction

Proinflammatory cytokines, including interleukin-1β (IL-1β) and tumor necrosis factor-α (TNF-α), play important roles as soluble mediators of innate immunity. In the central nervous system (CNS), cytokines contribute to diverse functions including tissue repair and synaptic activity. The brain is exposed to cytokines released from peripheral immune tissues and from the CNS itself. Microglia, the resident macrophages of the brain, are the main source of cytokines in the CNS. Cytokines released from activated microglia are known to stimulate astrocytes to secrete more inflammatory mediators as a secondary immune response. A characteristic feature of the inflammatory pathway is the feed-forward loop which leads to sustained release of cytokines. Chronic uncontrolled inflammation is known to contribute to neurodegeneration [1].

Cytokines act synergistically to cause neuronal injury through release of neurotoxic factors. For example, BDNF signaling and its neuroprotective actions are impaired by IL-1β in rat cortical neurons [2]. Exercise in Tg2576 mice leads to decreases in IL-1β and TNF-α in the brain and improved cognitive function [3]. IL-1β has been also shown to be a mediator of anti-neurogenic effects during chronic stress [4]. It is widely accepted that adult neurogenesis occurs throughout life. Neuroprogenitor cells persist in the subventricular zone of the lateral ventricles and subgranular layer of the dentate gyrus of the hippocampus of the adult brain [5], [6]. These self-renewing multipotent cells differentiate into neurons, astrocytes and oligodentrocytes. Recently, we developed a differentiation protocol to obtain ∼90% neurons from human neuroprogenitor cells by using a combination of agents including NGF, BDNF, dibutyryl cyclic AMP and retinoic acid [7]. These cells may prove to be a very good cell culture model to study the effects of cytokines and chemokines on neurogenesis.

Chemokines are a family consisting of ∼50 proteins that play important roles in cell migration and in neuroglial interactions [8]. They are divided into 4 groups, two of which are major classes, namely CC and CXC. Chemokines induce chemotactic response in cells expressing corresponding receptors [9]. Several studies have demonstrated that neurons express chemokines under physiological as well as pathological conditions [10]. Neuronal chemokines are involved in a variety of functions including differentiation, survival and synaptic transmission. In addition, they are essential for neuron-microglia and neuron-astrocyte communications. Sheng et al (2005) have reported TNF-α-mediated induction of the chemokines MCP-1 and CXCL10 in human neural precursor cells [11].

Altered signaling pathways during inflammation converge at the level of transcription factors leading to orchestrated gene expression patterns (reviewed in [12]). Cytokines are known to activate the transcription factors including NF-kB, AP-1 and STAT-1. As these transcription factors, in turn, induce more inflammatory mediators, there is a vicious cycle of sustained inflammation. The main objective of this study is to determine the molecular mechanism by which proinflammatory cytokines IL-1β and TNF-α exacerbate and sustain a chronic inflammatory state in differentiated human neuroprogenitor cells. We performed gene expression profiling of inflammatory mediators induced by IL-1β and TNF-α and examined the role of activated transcription factors. Bioinformatics analysis revealed novel network motifs involving multiple transcription factors including NF-kB, STAT-1, c-jun and C/EBPβ.

## Methods

### Reagents

Cell culture media and supplies were purchased from Gemini Bio Products, Inc. (Woodland, CA) and Invitrogen-Life Technologies (Rockville, MD). NPCs derived from human fetal brain at 9 weeks of gestational age were obtained as cryopreserved neurospheres from Lonza, Inc. (Walkersville, MD). Neurobasal medium, supplements for proliferation and differentiation of NPCs, epidermal growth factor (EGF) and fibroblast growth factor (FGF), were from Stemcell Technologies (Vancouver, BC, Canada). Poly-L-lysine, mouse laminin, DAPI and dibutyryl cyclic AMP were obtained from Sigma Chemical Co. (St. Louis, MO). IL-1β (50 U/ng) and TNF-α (100 U/ng) were from Roche Applied Science (Indianapolis IN, USA). Brain-derived neurotropic factor (BDNF) and antibodies directed against MAP2a, β tubulin, GFAP, phospho IKK, IKK, IkBα, phospho p65, p65, phospho STAT-1, STAT-1 and β-actin were from Cell Signaling (Beverly, MA). Abeta (1-42) peptide was purchased from Quality Control Biochemicals (QCB), Hopkinton, MA. Antibodies to IL-8 and CXCL10 were obtained from Santa Cruz Biotechnology (Santa Cruz, CA) and R & D systems (Minneapolis, MN) respectively. Anti-rabbit IgG and anti-mouse IgG conjugated to fluorescent probes (Cy3 or FITC) were from Jackson Immuno Research Laboratories (West Grove, PA).

### Culture and differentiation of human neuroprogenitor cells (NPCs)

**Figure 1:**
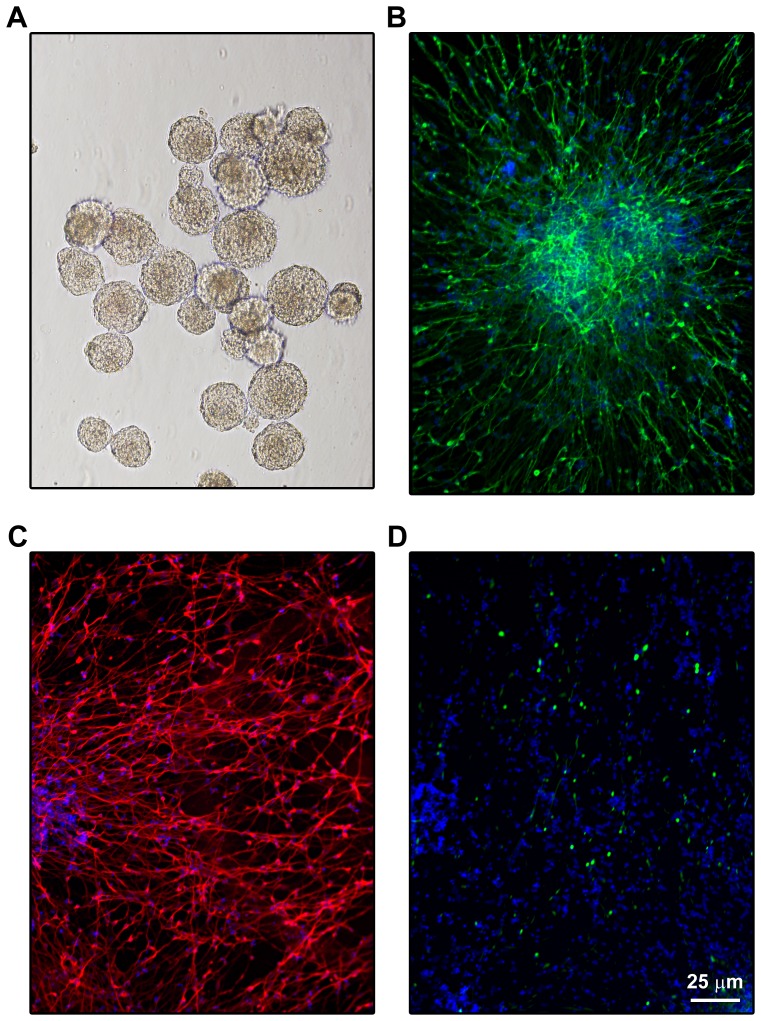
Human NPC-derived neuronal model. **A.** NPCs, derived from human fetal brain, were cultured and expanded as neurospheres in suspension. **B.** Four day-old neurospheres were seeded in dishes coated with poly-L-lysine and mouse laminin and cultured in the presence of 20 ng/ml NGF, 10 ng/ml BDNF, 100 µM dibutryl cyclic AMP and 1 µM retinoic acid for two wks. Differentiated NPCs were fixed and immunostained for neuronal markers MAP2a with FITC (**B** green) and β tubulin with cy3 (**C**; red) and for the glial marker, GFAP with FITC (**D**; green). Nuclei were stained blue with DAPI. The neuronal markers were observed in ∼90% of differentiated NPCs.

The NPCs were cultured in suspension as neurospheres (Fig. 1A) in neurobasal medium along with proliferation supplements, EGF (20 ng/ml) and FGF (20 ng/ml). When the neurospheres reached the size of >500 µm in diameter, they were transferred to 15-ml tubes and centrifuged at 1000 rpm for 5 min. The pellet was triturated 75 times to generate a single-cell suspension and the culture was continued to generate more neurospheres for upto 10 passages. Frozen stocks of partially expanded NPCs from different batches were revived and used for each set of experiments. NPCs have the potential to differentiate into neurons, astrocytes or oligodendrocytes after attachment to various substrata, depending on the culture condition. To obtain neuron-rich cultures, 4-day old neurospheres were seeded in 6-well dishes (coated with 100 µg/ml of poly-L-lysine, recoated with 5 µg/ml of mouse laminin and washed in PBS). The NPCs were cultured in the presence of 20 ng/ml NGF, 10 ng/ml BDNF, 100 µM dibutryl cyclic AMP and 1 µM retinoic acid. For immunofluorescence staining experiments, the NPCs were differentiated in 24-well dishes with coated coverslips.

### Preparation of Aβ oligomers and Aβ fibrils

The lyophilized Aβ peptide (1-42) was resuspended in hexafluoropropanol and incubated at RT for 1 h to monomerize the peptides. After evaporation of the solvent under vacuum, Aβ were stored at −20°C. Aβ oligomers were prepared by the method reported by Ryan et al (2010) that produces consistent soluble Aβ, stabilized by SDS [13]. Briefly, Aβ was redissolved in DMSO to 5 mM, bath-sonicated, diluted to 100 µM with cold PBS containing 0.05% SDS and vortexed (30 s). After initial aggregation at 4°C for 24 h, oligomers were diluted to 50 µg/ml and incubated at 4°C for 2 wks. Before use, the sample was centrifuged at 13K for 10 min at 4°C. Aβ fibril stock (100 µM) was prepared by incubation in HCl (10 mM) at 37°C for 24 h as in our previous report [14].

### Immunofluorescence staining of neurons

Differentiated NPCs were fixed in 4% paraformaldehyde for 30 min and washed twice in PBS. The cells were treated with permeabilization buffer (5% BSA and 0.2% Triton X PBS) for 90 min, and then incubated with primary antibodies, MAP2a (1∶200), β tubulin (1∶250), GFAP (1∶1000), IL-8 (1∶500), CXCL10 (1∶500), phospho p65 (1∶250) or STAT-1 (1∶250) overnight at 4°C in a humidified chamber [15]. After washing in PBS, cells were further incubated with anti-rabbit-Cy3, anti-mouse-FITC and DAPI (2 µg/ml; nuclear stain) at room temperature for 90 min. The cells were washed in PBS and the coverslips were placed upside down on slides over 10 µl of mounting medium, sealed with nail polish and examined by fluorescence microscopy.

### Human Inflammatory Cytokines & Receptors PCR Array

Total RNA was isolated from treated cells, using Versagene RNA isolation kit (Fisher Scientific, Pittsburgh, PA). RNA samples were treated with DNase and then converted to cDNA [16]. The cocktail for PCR array was prepared by adding 1278 µl of the RT^2^ qPCR SYBR Green master mix and 1173 µl H_2_0 to 102 µl of the diluted cDNA and 25 µl of this cocktail was added to each well of the 96-well PCR array plate (SABiosciences, Frederick, MD) containing primers for the 84 genes in human inflammatory pathway (listed in Table S1), 5 housekeeping control genes and 3 RNA and PCR quality controls as in our previous study [7]. Real-time PCR was performed with an ABI Prism 7700 sequence detector (Applied Biosystems, Foster City, CA). After amplification, real-time data acquisition and analysis were performed through the Data Analysis Web Portal (SA Biosciences). Data analysis is based on the delta-delta Ct method with normalization of the raw data to GAPDH as described in the manufacturer's manual.

### Custom-made Taqman low density array (TLDA)

TLDA in a 384-well microfluidic card allows simultaneous monitoring of real time PCR reactions. We generated a custom-made TLDA card in which the primers and Taqman probes are loaded in four sets. We selected genes in inflammatory pathways that were significantly modulated by IL-1β and TNF-α (Table 1). The cDNA was generated from DNase-treated RNA samples, combined with TaqMan Universal PCR master mix and loaded through the side ports as in our previous report [17]. The TLDA cards were briefly centrifuged to fill the wells and were run on the Applied Biosystems 7900HT Fast Real-Time PCR Systems. The expression of target genes was corrected for GAPDH levels.

**Table 1:** Human NPCs were differentiated into a neuron-rich culture for 2 weeks and incubated in the presence of 5 ng/ml each of IL-1β or TNF-α for 18 h.

	IL-1β	TNF-α		IL-1β	TNF-α
**CCL2**	83±14	117±16	**IL1A**	6.2±0.8	1.1±0.2
**CCL3**	2.7±0.4	1.9±0.1	**IL1B**	4.6±0.6	1±0.2
**CCL4**	1.2±0.3	2.2±0.2	**IL1F8**	4.4±0.6	1.5±0.2
**CCL5**	60±9.2	190±28	**IL1R1**	12±1.6	7.6±1.2
**CCL7**	79±12	7.5±11	**IL1RN**	5.6±0.7	5.7±0.8
**CCL8**	5.3±0.7	1.3±0.2	**IL5**	5.6±0.8	4.3±0.6
**CCL20**	5870±823	10716±1725	**IL8**	2376±318	483±62
			**IL10**	2.4±0.2	2.1±0.3
**CXCL1**	240±38	410±62	**IL13**	3±0.4	2.7±0.3
**CXCL2**	257±32	395±57	**IL13RA1**	4.2±0.4	7.3±0.9
**CXCL3**	205±28	133±19	**LTA**	3.4±0.4	4.9±0.6
**CXCL5**	212±34	261±42	**LTB**	2.1±0.2	5±0.7
**CXCL6**	118±18	117±21	**SPP1**	12±0.1.6	10±1.6
**CXCL9**	8.3±13	76±11	**TNF-α**	6.2±0.8	14±1.8
**CXCL10**	3666±437	14329±2318			
**CXCL11**	652±92	3189±474			
**CXCL12**	2.4±0.3	3.0±0.4	**C3**	226±34	441±58
**CXCL14**	4.5±0.5	7.9±1.2	**C4A**	2.4±0.2	3.5±0.4
			**BCL6**	4.4±0.5	4.4±0.5
CCR7	55±7.2	18±2.6	C/EBPβ	17±2.5	21±3.5

Induction of inflammatory mediators by IL-1β and TNF-α in differentiated human NPCs (Mean fold induction). RNA was isolated and an immune pathway-specific array was performed. Values represent mean fold induction over untreated control from four independent experiments. More than 100-fold inductions are shown in blue.

### CXCL10 promoter analysis by transient transfection

The following promoter constructs of CXCL10 linked to firefly luciferase reporter were provided by Dr. Richard Ransohoff [18]. 1. Truncated promoter of CXCL10 containing ISRE and kB sites. 2. Truncated CXC10 promoter with mutated ISRE site. 3. Truncated CXCL10 promoter with mutated kB site. Transient transfections in differentiated human NPCs were carried out using LipofectAMINE™2000 reagent (Invitrogen-Life Technologies, Carlsbad, California, USA) [19]. A constitutively active renilla luciferase (pRL-TK-luc) was included to correct for transfection efficiency. After 6 hours, the transfected cells were exposed to cytokines for 18 hours. Luciferase activity was measured in the cell lysates using a dual luciferase assay kit (Promega, Madison, WI, USA).

### Western blot analysis

Differentiated human NPCs, following treatments, were lysed with mammalian protein extraction buffer (Pierce, Rockford, IL), supplemented with phosphatase and protease inhibitors [7]. The protein content was determined [20] in the supernatant (12,000 RPM) of cell lysates. The proteins were resolved on a 12% SDS-PAGE and then transferred to PVDF membranes. The blots were blocked in 5% nonfat dry milk at RT for 1 h and then incubated in the presence of primary antibodies (1∶1000) at 4°C. After incubation with secondary antibodies conjugated to alkaline phosphatase, signals were developed with CDP-Star reagent (New England Biolabs, Beverly, MA). The intensity of bands was measured using Fluor-S MultiImager and Quantity One software from Bio-Rad corrected for the levels of β actin.

### Statistical analysis

Data presented are Mean ± SE of 3–4 independent experiments. Statistical evaluation was performed by one-way ANOVA with Dunnett's multiple comparison test.

### Bioinformatics analysis

Network motif (NM) analysis was performed as previously described [21]. The protein-protein interactions (PPI) were extracted from human protein reference database (HPRD) [22]. The human protein-DNA interaction (PDI) data were obtained from TRANSFAC database [23]. These data sets consist of 20,473 pairs of PPIs and 2546 pairs of PDIs. The NM analysis was performed in FANMOD software tool [24]. All connected subnetworks containing three nodes (i.e., genes) in the gene regulatory network (GRN) were collated into isomorphic patterns, and the number of times each pattern occurred was counted. If the number of occurrences is at least five and significantly higher than in randomized networks, the pattern was considered as a NM. The statistical significance test was performed by generating 1000 randomized networks and computing the fraction of randomized networks in which the pattern appeared at least as often as in the interaction network, as described in detail in Yeger-Lotem *et al.* [25]. A pattern with P value of <0.05 was considered statistically significant.

## Results

### Human neuroprogenitor cells (NPCs)-derived neuronal model

Human NPC-derived neurons provide an excellent cell culture model to study the mechanism of neurodegeneration. The major advantages of this model are that NPCs can be expanded for up to 10 passages to provide abundant supply of cells (Fig. 1A) and differentiated cells display the features of primary neurons. We previously reported a differentiation protocol to obtain neuron-rich cultures [26]. Subsequently, we have further improved the procedure to obtain neuronal cultures consistently [7]. For example, we have introduced the step of seeding 4-day old neurospheres instead of single cell suspension in dishes to enable contacts between differentiating neurons. In addition, 4–5 days after seeding, differentiation supplement (Stem cell technologies) is replaced with B27 supplement (Invitrogen). Immunofluorescent staining for neuronal markers including MAP2a (Fig. 1B) and β tubulin (Fig. 1C) showed ∼90% of differentiated NPCs to be neuronal in nature. GFAP, an astrocyte marker, was detected in <10% of the cells (Fig. 1D). By increasing the concentration of retinoic acid from 2 µM to 5 µM, we were able to reduce the population of astrocytes further (results not shown). However, decreasing the astrocyte population reduces the long-term survival of neurons. Therefore such manipulations were introduced, 3–5 days before performing the experiment.

### Induction of inflammatory mediators by IL-1β and TNF-α

IL-1β and TNF-α are among the cytokines secreted by activated microglia. These cytokines in turn can act on neurons and astrocytes to induce the expression of more cytokines and chemokines leading to an inflammatory loop. To investigate this secondary immune response, an inflammation pathway-specific gene expression profiling was performed with differentiated NPCs exposed to IL-1β and TNF-α. Robust induction of several chemokines was observed following exposure to the cytokines (Table 1). Induced chemokines in the CCL family were CCL2 (MCP-1), CCL3 (MIP-1α), CCL4 (MIP-1β), CCL5 (RANTES), CCL7 (MCP-3), CCL8 (MCP-2) and CCL20 (MIP-3α). In the CXCL family, CXCL1, CXCL2, CXCL3, CXCL5, CXCL6, CXCL9 (Mig), CXCL10 (IP-10), CXCL11 (I-TAC), CXCL12 and CXCL14 were induced. Among 11 chemokine receptors examined, we observed the induction of CCR7 alone. Several cytokines including IL-1β, TNF-α, IL-5, IL-10 and IL-13 were induced modestly by 3–6 folds. Strong induction of IL-8, a cytokine with known apoptotic effects, suggests that the inflammatory loop triggered by IL-1β and TNF-α could potentially be a cause of neuronal death. IL-1β and TNF-α strongly induced the compliment factor C3 which is known to play a role in neuroinflammation in the Alzheimer's brain. Complement system is an effector mechanism of the inflammatory pathway in clearing deleterious substances. Protective role of C3 in Aβ removal has been reported [27]. We also observed increases in the mRNA levels of c/EBPβ, a transcription factor known to induce inflammatory genes. Thus exposure of neurons to IL-1β and TNF-α could set in motion complex transcriptional regulatory pathways leading to amplification of inflammatory response.

### Network motifs (NMs) involved during the induction of inflammatory mediators

**Figure 2:**
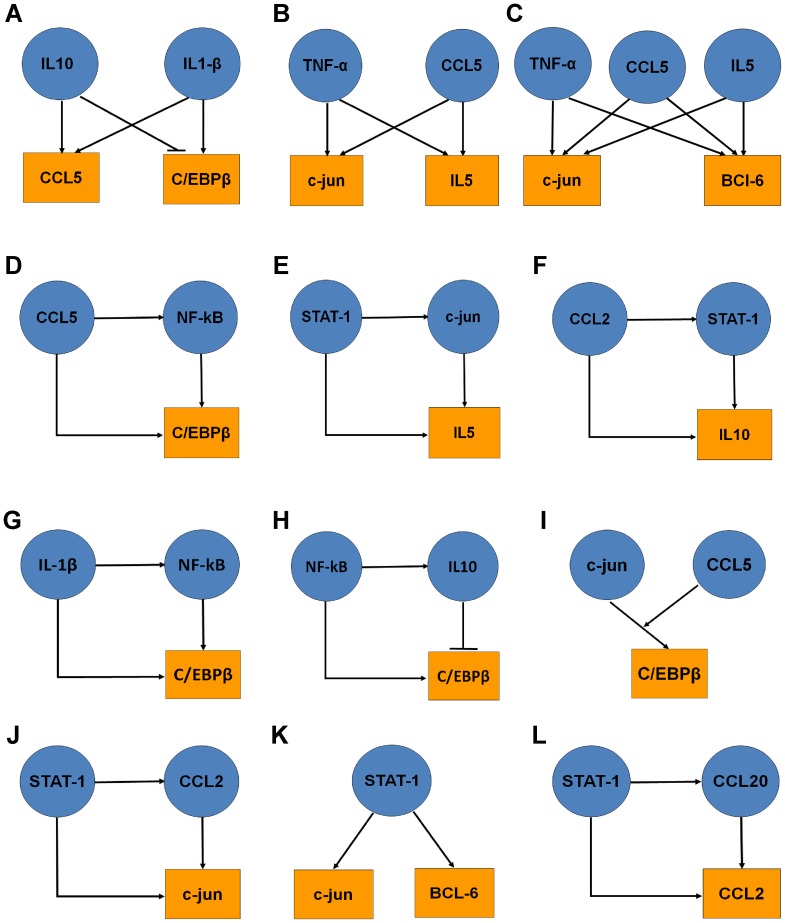
Network motifs (NMs) involved during the induction of inflammatory mediators. From the list of inflammatory genes induced in differentiated human NPCs exposed to IL-1β and TNF-α, protein-protein and protein-DNA interactions were extracted from appropriate databases. NM analysis was performed using FANMODE tool. Representative NMs (A-L) from 118 identified are presented. Multi-input motifs (Fig. 2A–Fig. 2C) and feed-forward loops (Fig. 2D–Fig. 2G) were observed.

Network motif (NM) analysis with the immune array data using FANMOD software [24] identified 118 significant NMs (table S2). Seventeen genes in the array appeared in more than 10 NMs, suggesting complex regulatory interactions during immune response. One or more transcription factors were involved in 40 NMs (table S3). In this study, we focused on three-node NMs, because larger size NMs (>3 nodes) were composed of three-node ones in most cases. Representative NMs including multi-input motifs (A–C) and feed-forward loops (D–G) are shown in figure 2. They are likely to amplify and exacerbate the inflammatory pathway. Feed-back inhibition was also observed in the case of the antiinflammatory cytokine IL10 and C/EBPβ (Fig. 2H).

### Cytokine-mediated elevation of IL-8 and CXCL10 proteins in differentiated NPCs

**Figure 3:**
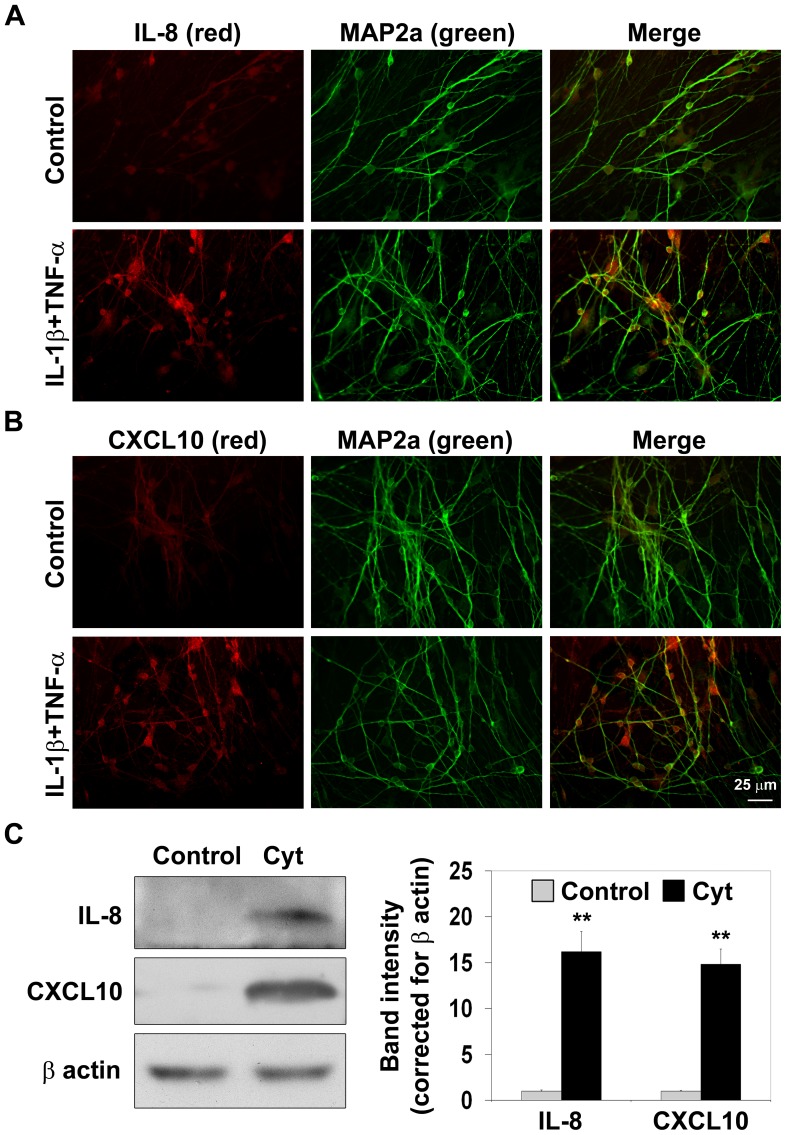
Cytokine-mediated elevation of IL-8 and CXCL10 proteins. Human NPCs, differentiated for 2 weeks, were preincubated with Golgi plug (1 µl/ml), a secretion inhibitor and then exposed to a combination of 5 ng/ml each of IL-1β and TNF-α for 18 h. Treated cells were fixed and immunostained for MAP2a with FITC (green) and for IL-8 (**A**) or CXCL10 (**B**) with cy3 (red). Cytokine-mediated increases in the levels of IL-8 and CXCL10 were observed in MAP2a-positive cells. **C.** Differentiated NPCs exposed to IL-1β and TNF-α (Cyt) were processed for the Western blot analysis of IL-8, CXCL10 and β actin. Representative images are presented. The band intensities of IL-8 and CXCL10 were determined by scanning and corrected for the levels of β actin. The results are M±SE of three independent observations. **P<0.001 compared to untreated control.

Immunofluorescence staining was performed to examine IL-8 and CXCL10, two strongly induced inflammatory mediators, at the protein level. The NPCs, differentiated for 2 wks, were treated with GolgiPlug, a secretion inhibitor for intracellular accumulation of inflammatory mediators. Increased levels of IL-8 and CXCL10 in MAP2a-positive neurons were observed following exposure to a combination of IL-1β and TNF-α, suggesting neuron-specific expression (Fig. 3A and Fig. 3B). As expected, the chemokine levels were elevated in nonneuronal cells as well. Western blot analysis showed significant increases (P<0.001) in the protein levels of IL-8 and CXCL10 (Fig. 3C). Although we have presented findings with a combination of IL-1β and TNF-α to get strong signals for IL-8 and CXCL10, we did observe increases in the levels of these targets following treatment with individual cytokines (results not shown).

### Role of NF-kB and STAT-1 in the induction of inflammatory response

Cytokines are known to activate the transcription factors NF-kB and STAT-1. To determine their role in the induction of inflammatory genes by IL-1β and TNF-α, neurons were preincubated with Bay 70-1185, an inhibitor of NF-kB and a JAK inhibitor to block STAT-1 activation. A custom-made Taqman low density array (TLDA) was performed to examine the induction inflammatory mediators, selected from the findings of inflammatory pathway array (Table 1). Inhibition of signaling to NF-kB and STAT-1 resulted in 30–50% decreases in the basal level expression of inflammatory mediators (Table 2). Inhibition of NF-kB led to decreased induction of cytokines and chemokines by IL-1β (30–90%) and TNF-α (45–90%). Blocking STAT-1 action with a JAK inhibitor resulted in decreased induction of all chemokines except CXCL2. Overall, inhibitor studies suggested that IL-1β action is predominantly through NF-kB with STAT-1 playing a minor role. In the case of TNF-α, both transcription factors played equal roles. In addition, IL-1β and TNF-α showed synergistic actions in the induction of CCL-5, CXCL10, CXCL11, IL-8 and C3 and additive actions with respect to CCL-2, CCL3, CCL20, CXCL2 and CXCL9.

**Table 2:** Differentiated human NPCs were exposed to 5 ng/ml of IL-1β or TNF-α in the absence or presence of 15 µM of Bay 11-7085, NF-kB inhibitor (Bay) or 1 µM of JAK inhibitor (JI) to block STAT-1 activation.

	Control	IL-1β	TNF-α	IL-1β+TNF-α
**CCL2**	1	31.5±4.6	77.6±9.2	112±12
**CCL3**	1	2.3±.3	2.6±.3	6.2±0.5
**CCL5**	1	12.5±1.7	192±24	403±52*
**CCL20**	1	186±26	107±16	312±37
**CXCL2**	1	21.6±3.7	30.2±3.1	54.3±7.4
**CXCL9**	1	8.3±1.2	27.4±3.6	31.4±4.1
**CXCL10**	1	164±25	595±74	977±117*
**CXCL11**	1	53.4±8.3	235±39	435±51*
**IL8**	1	178.3±28	117±14	454±48*
**C3**	1	18.1±2.9	36.2±42	82.4±11*

	Control	IL-1β	Bay+IL-1β	JI+ IL-1β	TNF-α	Bay+TNF-α	JI+TNF-α
**CCL2**	1	31.5±4.6	22.1±3.1	23.9±3.2	77.6±9.2	33.5±4.9	50.8±6.2
**CCL3**	1	2.3±.3	1.5±0.2	1.7±0.2	2.6±.3	1.4±0.2	1.6±0.1
**CCL5**	1	12.5±1.7	6.3±0.8	9.4±1.3	192±24	34.5±4.8	45.7±5.3
**CCL20**	1	186±26	22.3±3.8	108±12.1	107±16	11.2±0.9	38.6±4.1
**CXCL2**	1	21.6±3.7	13.7±2.2	20.3±2.6	30.2±3.1	13.2±1.5	27.4±3.7
**CXCL9**	1	8.3±1.2	3.2±0.3	6.5±0.7	27.4±3.6	6.5±0.8	8.2±1.3
**CXCL10**	1	164±25	11.4±1.6	73.4±8.3	595±74	77.2±9.1	80.3±8.9
**CXCL11**	1	53.4±8.3	9.3±1.2	17.4±2.4	235±39	45.7±5.1	24.1±3.4
**IL8**	1	178±28	53±6.1	146±16.1	117±14	30.4±3.7	21.6±3.6
**C3**	1	18.1±2.9	4.4±0.5	4.9±0.7	36.2±42	9.5±1.2	6.2±0.7

Induction of cytokines and chemokines by IL-1β and TNF-α involves activation of NF-kB and STAT-1 (Mean fold induction).

Cells were also exposed to a combination of IL-1β and TNF-α. After 18 h, the neurons were processed for RNA isolation and custom-made Taqman low density array (TLDA). Values represent mean fold induction over untreated control from four independent arrays. Cytokine-mediated induction of inflammatory mediators involved activation of STAT-1 and NF-kB.

* Synergistic induction by IL-1β and TNF-α.

### Cytokine-mediated activation of signaling to NF-kB and STAT-1

**Figure 4:**
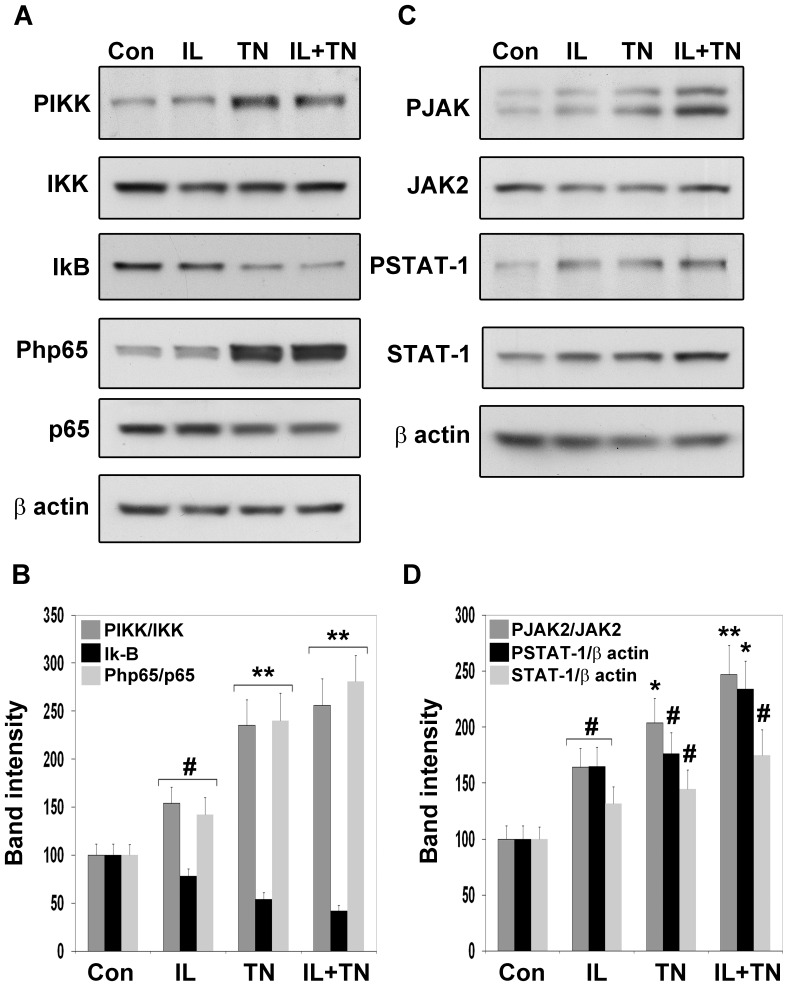
Cytokine-mediated activation of signaling to NF-kB and STAT-1. Differentiated NPCs were exposed to 5 ng of IL-1β and TNF-α, alone or in combination, for 2 h and processed for the Western blot analysis of the upstream kinases of NF-kB (A and B) and STAT-1 (C and D) pathways. Band intensities were quantitated by scanning. Increased phosphorylation of IKK (PIKK) and p65 (Pp65) was observed, especially in TNF-α-treated cells with IL-1β showing modest actions. Both cytokines showed additive actions in the phosphorylation of JAK2 (PJAK2) and STAT-1 (PSTAT-1). The results are M±SE of three independent observations. #P<0.05, *P<0.01 and **P<0.001 versus untreated control.

NF-kB is sequestered in the cytoplasm by the inhibitor IkB. IKK-mediated phosphorylation of IkB leads to its proteosomal degradation resulting in the nuclear localization of NF-kB. In addition, p65, a member of NF-kB family in neurons, is known to be phosphorylated at serine 536 which facilitates NF-kB-dependent gene expression [28]. Western blot analysis showed 45–55% (P<0.05) increase in the active phosphorylated form of IKK and p65 and 23% (P<0.05) decrease in the level of IkB following treatment with IL-1β (Fig. 4A and 4B). TNF-α, on the other hand was a strong inducer of the NF-kB pathway with 140% (P<0.001) increase in the phosphorylation of IKK and p65 and 46% (P<0.001) decrease in the level of IkB. The combination of these cytokines did not have any additive effects. STAT-1 is known to be activated by JAK2-mediated phosphorylation, following which it translocates to the nucleus. TNF-α and IL-1β modestly activated the JAK/STAT pathway when added alone to the differentiated NPCs whereas a combination of these cytokines had additive effects (Fig. 4C and D). They increased the active phosphorylated form of JAK2 by 145% (P<0.001) and STAT-1 phosphorylation by 130% (P<0.01). The levels of STAT-1 also increased significantly (P<0.05) as the expression of this transcription factor itself is induced.

### Cytokine-mediated nuclear localization of NF-kB and STAT-1

**Figure 5:**
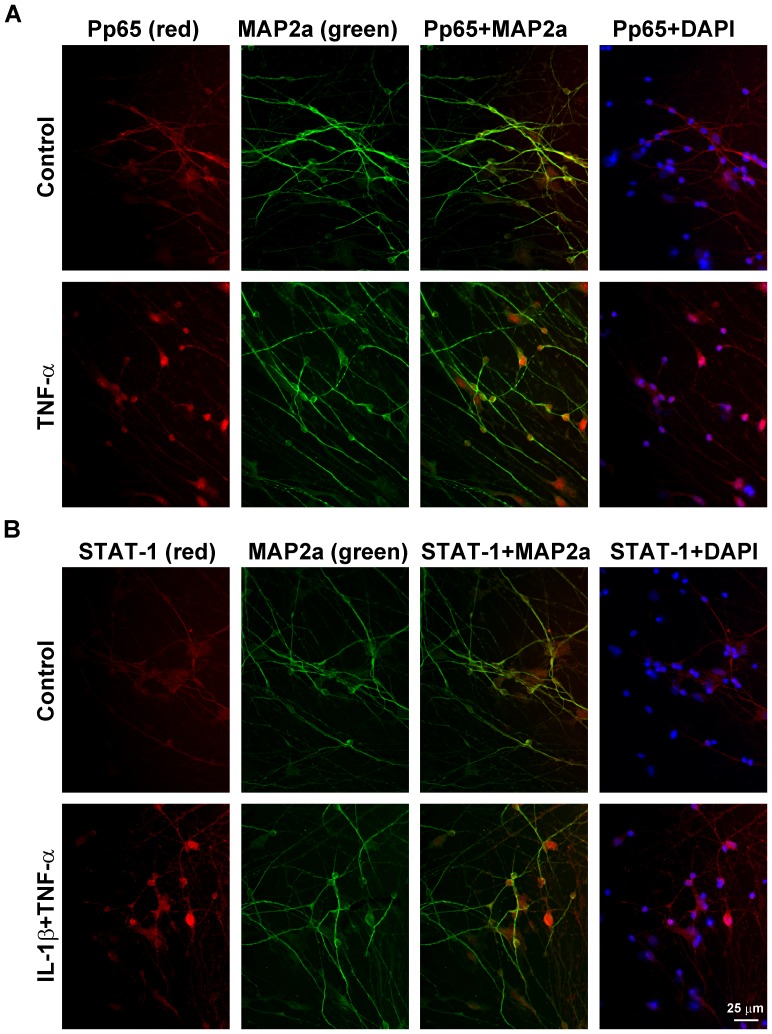
Cytokine-mediated nuclear localization of NF-kB and STAT-1. Differentiated NPCs were treated with TNF-α (**A**; 5 ng/ml) or a combination of IL-1β and TNF-α (**B**; 5 ng/ml each) for 6 h, fixed and immunostained for the phosphorylated form of p65 (Pp65; **A**), a member of NF-kB family or STAT-1 (**B**) with cy3 (red) and for MAP2a with FITC (green). Nuclear localization of phospho p65 and STAT-1 was observed following exposure to TNF-α and a combination of IL-1β and TNF-α respectively. Representative images from three independent experiments are presented.

To further confirm the activation of NF-kB and STAT-1 in cytokine-treated differentiated NPCs, we examined the nuclear localization of these transcription factors by immunofluorescence staining. Because Western blot analyses showed TNF-α to be a stronger inducer of NF-kB, the cells were exposed to this cytokine alone for this transcription factor. Cy3 (red)-stained signal for phospho p65 colocalized with MAP2a-positive neurons as well as DAPI (blue)-stained nuclei suggesting neuronal nuclear localization following exposure to TNF-α (Fig. 5A). IL-1β showed modest action on the nuclear localization of p65 (results not shown). Because of the additive actions of IL-1β and TNF-α on JAK/STAT pathway (Fig. 4C), a combination of these cytokines was used to examine the nuclear localization of STAT-1. Translocation of STAT-1 to the nucleus in neurons was observed following exposure to cytokines (Fig. 5B). Nuclear localization of phospho p65 and STAT-1 were observed in MAP2a-negative glial cells as well.

### Induction of CXCL10 promoter by IL-1β and TNF-α

**Figure 6:**
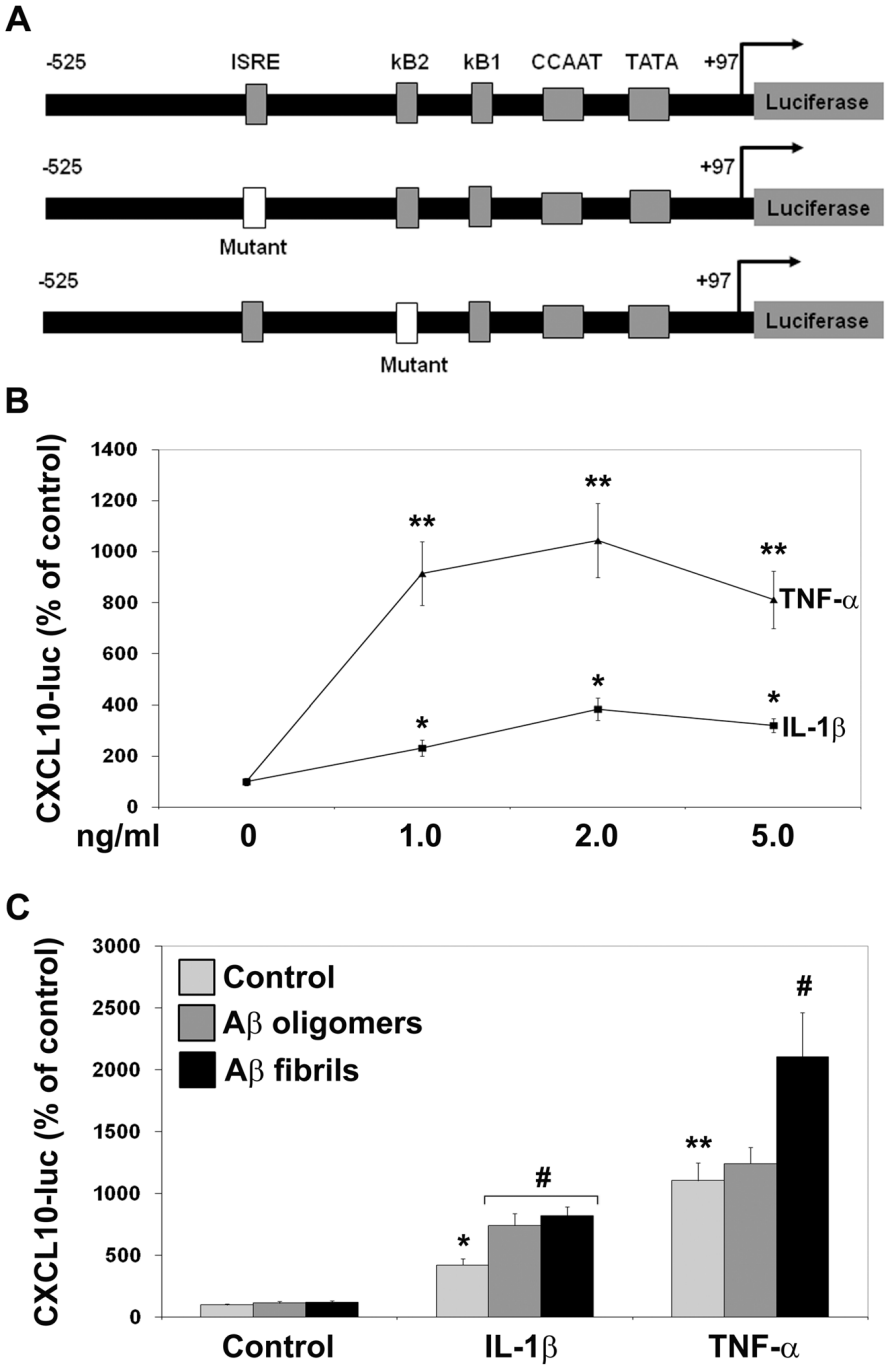
Induction of CXCL10 promoter by IL-1β and TNF-α. **A.** Truncated wild type, ISRE mutant and kB mutant CXCL-10 promoter constructs linked to a firefly luciferase reporter gene were used in the transient transfection assay. **B.** Human NPC-derived neurons were transfected with wild type CXCL10 promoter along with a constitutively active renilla luciferase reporter and exposed to increasing concentrations of IL-1β and TNF-α. The cytokines were also added to transfected neurons in the absence and presence of 2 µM of Aβ oligomers or Aβ fibrils (**C**). After 18 h, the treated neurons were processed for the assay of luciferases. The ratios of firefly and renilla luciferase were taken as measures of promoter activity. TNF-α was a stronger inducer of CXCL10 compared to IL-1β. *P<0.01; **P<0.001 compared to untreated control. #P<0.01 vs cells treated with cytokines in the absence of Aβ aggregates.

CXCL10 (IP-10) is a chemokine known to be induced in the Alzheimer's brain [29]. We had observed strong induction of CXCL10 in differentiated NPCs by IL-1β and TNF-α (Table 1 and Figure 2). Therefore we chose the promoter for this chemokine for additional mechanistic studies. CXCL10 promoter has been reported to contain response elements for NF-kB and STAT-1 [18]. The wild type promoter and the constructs in which ISRE or kB sites are mutated, linked to a luciferase reporter gene are shown in figure 6A. When neurons were transfected with the wild type promoter and then exposed to the cytokines, 10-fold induction was observed at a concentration of 2 ng/ml of TNF-α. IL-1β induced the promoter by 4-fold at the same concentration (Fig. 6B). Aβ oligomers and Aβ fibrils did not have any direct effect on CXCL10 promoter activity. However, IL-1β action was enhanced by both oligomers and fibrils and TNF-α action was enhanced by fibril alone (Fig. 6C).

### Cytokine-mediated induction of CXCL10 promoter requires NF-kB and STAT-1

**Figure 7:**
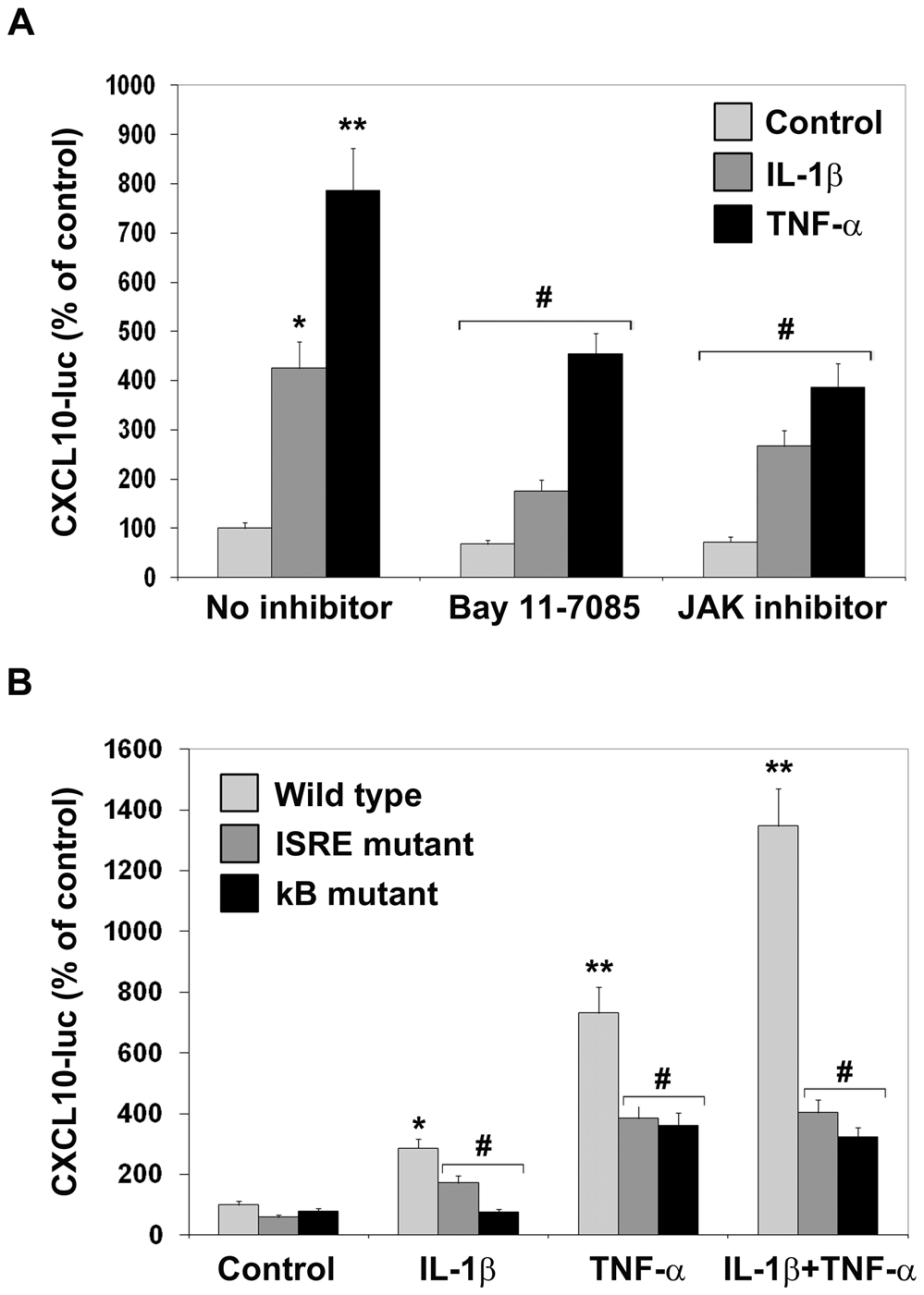
Cytokine-mediated induction of CXCL10 promoter requires NF-kB and STAT-1. **A.** Differentiated NPCs transfected with wild type CXCL10 promoter were preincubated in the presence of 10 µM of Bay 11-7085, NF-kB inhibitor or 1 µM of JAK inhibitor to block STAT-1 activation, followed by exposure to 2 ng/ml each of IL-1β and TNF-α for 18 h. **B.** Cells were transfected with wild type, and mutant promoter constructs and cultured in the presence of 2 ng/ml each of IL-1β, TNF-α or both for 18 h. Treated cells (**A** and **B**) were processed for the assay of luciferases. Inhibition of NF-kB or STAT-1 resulted in significant decreases in cytokine-mediated promoter activation. Mutation of ISRE or kB elements led to loss of synergy between IL-1β and TNF-α. *P<0.01; **P<0.001 compared to untreated control. #P<0.01 vs cells treated with cytokines in the absence of inhibitors (**A**) or cells transfected with wild type promoter (**B**).

Inhibition of NF-kB with Bay 11-7085 and inhibition of STAT-1 with JAK inhibitor resulted in significant (P<0.01) decreases in promoter induction by IL-1β and TNF-α, suggesting a role for both transcription factors in the induction of CXCL10 (Fig. 7A). Mutations of ISRE or kB sites in the promoter resulted in similar decreases in basal as well as cytokine-induced CXCL-10 promoter activity (Fig. 7B). In addition, mutations led to loss of synergy between IL-1β and TNF-α. For example, in the case wild type promoter, the activity increased by 1250% in the presence of combination of IL-1β and TNF-α which is more that the additive value of increases by IL-1β (186%) and TNF-α (732%). Similar synergistic effect was not observed when either site was mutated.

## Discussion

Chronic uncontrolled inflammation has been shown to be associated with neurodegeneration [1], [30]–[33]. Although activated microglia are the main source of inflammatory mediators, astrocytes and neurons are also known to produce cytokines and chemokines [10]. This secondary immune response can lead to a vicious cycle and sustain a chronic inflammatory state. In this study, we demonstrate robust induction of a large number of inflammatory mediators in differentiated human NPCs, exposed to IL-1β and TNF-α, by a mechanism involving several transcription factors including NF-kB and STAT-1. Network motif-based analysis revealed complex interactions between transcription factors and their target genes.

The neuronal model used in this study is derived from human neuroprogenitor cells (NPCs). Our differentiation protocol yielded consistently a neuron-rich population that was maintained in culture for upto 10 wks [7]. IL-1β and TNF-α induced an array of inflammatory mediators in these cells as shown by pathway-specific gene expression profiling (Table 1). TNF-α was a stronger inducer compared to IL-1β with respect to majority of targets. Together, these two cytokines showed additive or synergistic actions. Positive feedback loop was a characteristic feature of this response. Induction of C/EBPβ, another inflammatory transcription factor, suggests a cascade of events that is likely to play an important role in chronic inflammation. Bioinformatics analysis was performed to determine gene regulatory network (GRN) among the induced genes. GRN is a type of biological network that describes the interactions between the transcription factors and their downstream genes. The GRNs are made up of repeated occurrences of simple patterns, called network motifs (NMs) [34]. Each NM performs a defined information processing function within the network. Same NMs have been discovered in diverse organisms from bacteria to human. The underlying hypothesis is that the NMs were independently selected by evolutionary processes with their characteristic dynamical functions and serve as building blocks in GRNs that are beneficial to the organism. In this study, we identified 118 NMs following treatment of neurons with a combination of IL-1β and TNF-α. Multi-input motifs (Fig. 2A–C) and feed-forward loops (Fig. 2D–G) are likely to play critical roles in the amplification of immune response. Another significant finding from the NM analysis was the identification of cross-talk between C/EBPβ and NF-kB.

Although the main objective of this study was to determine the molecular mechanism of the inflammatory loop induced by IL-1β and TNF-α, an additional important finding was the neuron-specific expression of inflammatory mediators which is in agreement with several reports (reviewed in [10]). A general understanding in the field of neuroinflammation is that neurons are passive target cells that get injured by cytokines secreted from microglia and astrocytes. However, several studies have reported that neurons produce inflammatory mediators as a stress response when injured [35]–[37]. For example, interleukin-6 expression is induced in axotomized sensory neurons [36]. Neuronal production of TNF-α in the rodent brain has been reported following ischemia [35] and isoflurane anesthesia [38]. Neuron-specific expression of TNF-α in the transgenic Alzheimer's mouse brain has been shown to enhance the inflammatory environment [39]. Injured neurons could send out distress signals in the form of chemokines to recruit microglia to the site of injury. We demonstrate in the current study that increases in the levels of IL-8 and CXCL10 are seen in MAP2a-positive neurons following an inflammatory stimulus. Dual immunofluorescent staining also showed nuclear localization of p65 and STAT-1 in neurons.

Induction of CXCL10 in the Alzheimer's brain, especially in the neurons of cortex and hippocampus, has been reported [29]. CXCL10 colocalizes with β amyloid deposits. CXCL-10 secreted from neurons and astrocytes can play an important role in the recruitment of microglial cells that express the corresponding receptor, CXCR3. The role of CXCL10/CXCR3 signaling in neuronal death has been suggested by the observation that neuronal death is diminished in CXCL10^−/−^ and CXCR3^−/−^ mouse brain [40]. Neuronal apoptosis in spinal cord injury is inhibited when CXCL10 is blocked with an antibody [41]. Because of the important role played by this chemokine in CNS pathologies, we took a closer look at its regulation at the promoter level. TNF-α was found to be a stronger inducer of CXCL10 promoter when compared to IL-1β (Figure 6B). Aβ aggregates did not induce CXCL10 in differentiated human NPCs but enhanced cytokine action (Fig. 6C). Both cytokines showed synergy at a concentration of 2 ng/ml which was lost when ISRE or kB response elements were mutated (Fig. 7B). Thus our findings suggest that synergistic actions of cytokines through NF-kB and STAT-1 could play an important role in the exacerbation of inflammatory response. We have previously reported the induction of CXCL10 in human islets by proinflammatory cytokines through activation of NF-kB and STAT-1 [42]. CXCL10 is involved in the recruitment of immune cells in islets and in β cell failure [43], [44].

Although the current study focused on NF-kB and STAT-1, involvement of other transcription factors in the induction of inflammatory mediators cannot be ruled out. Previous studies have demonstrated the role of c-jun/AP-1 in neuroinflammation [1]. For example, the expression of inflammatory genes in the brain endothelial cells is mediated by JNK-AP1 pathway but not through NF-kB [45]. CXCL10 expression in microglia involves activation of p38MAPK and JNK pathways, suggesting cell type-dependent transcriptional mechanism [46]. Very limited information is available on the role of STAT-1 in inducing inflammatory mediators in the brain. The current study suggests that STAT-1 is essential for the synergistic actions of IL-1β and TNF-α. Inhibition of JAK/STAT pathway resulted in 40–80% inhibition in the induction of several chemokines by TNF-α and modest inhibition of IL-1β action. STAT-1 is also known to mediate cytokine-induced apoptosis [47], [48] in pancreatic β cells. A similar pathway could cause neuronal death during neurodegeneration. We did observe a significant decrease in the induction of IL-8, a proapoptotic cytokine, following inhibition of JAK-STAT pathway. Thus our present study suggests that STAT-1 is a potential therapeutic target to break the synergy between cytokines that leads to sustained chronic neuroinflammation.

## Conclusions

Microglia-generated IL-1β and TNF-α can trigger a secondary immune response in astrocytes and neurons leading to an inflammatory loop that sustains a chronic inflammatory state in the brain. Such downstream effects of microglia activation, at some point, can become independent of the original trigger that initiated the process of inflammation. In this study, we demonstrate that the proinflammatory cytokines IL-1β and TNF-α induce an array of chemokines and cytokines in differentiated human neuroprogenitor cells. The involvement of multiple transcription factors including NF-kB and STAT-1, leading to a cascade, was shown by a novel network motif-based analysis. Findings from this study suggest that targeting the synergy between transcription factors induced by cytokines might eventually become an effective antiinflammatory treatment.

## Supporting Information

Table S1 **Functional gene grouping of inflammatory cytokines and receptors array**
(DOCX)

Table S2 **Network motifs (NMs) involved during the induction of an inflammatory loop by IL-1β and TNF-α in differentiated human neuroprogenitor cells.** Based on protein-protein and protein-DNA interactions, extracted from appropriate databases, NM analysis was performed using FANMODE tool. Identified 118 NMs are listed.
(DOCX)

Table S3 **Network motifs (NMs) with a transcription factor.** Identified 40 NMs with more than one transcription factor are listed.
(DOCX)
