# Supplementary material for: Induction of an Inflammatory Loop by Interleukin-1β and Tumor Necrosis Factor-α Involves NF-kB and STAT-1 in Differentiated Human Neuroprogenitor Cells
Source: PLoS One. 2013 Jul 29;8(7):e69585. doi: 10.1371/journal.pone.0069585 (PMC3726669; doi:10.1371/journal.pone.0069585)
Supplement: Table S1 — Functional gene grouping of inflammatory cytokines and receptors array (DOCX) [file pone.0069585.s001.docx]

**Supplementary table S1: Functional gene grouping of inflammatory cytokines and receptors array**

**Chemokine Genes:** C5, CCL1 (I-309), CCL11 (eotaxin), CCL13 (mcp-4), CCL15 (MIP-1d), CCL16 (HCC-4), CCL17 (TARC), CCL18 (PARC), CCL19, CCL2 (mcp-1), CCL20 (MIP-3a), CCL21 (MIP-2), CCL23 (MPIF-1), CCL24 (MPIF-2 / eotaxin-2), CCL25 (TECK) , CCL26, CCL3 (MIP-1a), CCL4 (MIP-1b), CCL5 (RANTES), CCL7 (mcp-3), CCL8 (mcp-2), CXCL1, CXCL10 (IP-10), CXCL11 (I-TAC / IP-9), CXCL12 (SDF1), CXCL13, CXCL14, CXCL2, CXCL3, CXCL5 (ENA-78 / LIX), CXCL6 (GCP-2), CXCL9, IL13, IL8.

**Chemokine Receptors:** CCL13 (mcp-4), CCR1, CCR2, CCR3, CCR4, CCR5, CCR6, CCR7, CCR8, CCR9, CX3CR1, IL8RA, XCR1 (CCXCR1).

**Cytokine Genes:** CD40LG (TNFSF5), IFNA2, IL10, IL13, IL17C, IL1A, IL1B, IL1F10, IL1F5, IL1F6, IL1F7, IL1F8, IL1F9, IL22, IL5, IL8, IL9, LTA, LTB, MIF, SCYE1, SPP1, TNF.

**Cytokine Receptors:** IFNA2, IL10RA, IL10RB, IL13, IL13RA1, IL5RA, IL9, IL9R.

**Other Genes Involved in Inflammatory Response:** ABCF1, BCL6, C3, C4A, CEBPB, CRP, ICEBERG, IL1R1, IL1RN, IL8RB, LTB4R, TOLLIP.
