# Supplementary material for: Induction of an Inflammatory Loop by Interleukin-1β and Tumor Necrosis Factor-α Involves NF-kB and STAT-1 in Differentiated Human Neuroprogenitor Cells
Source: PLoS One. 2013 Jul 29;8(7):e69585. doi: 10.1371/journal.pone.0069585 (PMC3726669; doi:10.1371/journal.pone.0069585)
Supplement: Table S2 — Network motifs (NMs) involved during the induction of an inflammatory loop by IL-1β and TNF-α in differentiated human neuroprogenitor cells. Based on protein-protein and protein-DNA interactions, extracted from appropriate databases, NM analysis was performed using FANMODE tool. Identified 118 NMs are listed. (DOCX) [file pone.0069585.s002.docx]

**Supplementary table S2. Network motifs (NMs) involved during the induction of an inflammatory loop by IL-1β and TNF-α in differentiated human neuroprogenitor cells.** Based on protein-protein and protein-DNA interactions, extracted from appropriate databases, NM analysis was performed using FANMODE tool. Identified 118 NMs are listed.

|  | **Network Motif ID** | **Node 1** | **Node 2** | **Node 3** |
| --- | --- | --- | --- | --- |
| 1 | 220 | 'CEBPB' | 'CCL5' | 'IL10' |
| 2 | 220 | 'CEBPB' | 'CCL5' | 'il1b' |
| 3 | 220 | 'CEBPB' | 'IL10' | 'il1b' |
| 4 | 220 | 'IL5' | 'cjun' | 'TNFa' |
| 5 | 220 | 'IL5' | 'cjun' | 'CCL5' |
| 6 | 220 | 'cjun' | 'TNFa' | 'CCL5' |
| 7 | 3230 | 'BCL6' | 'cjun' | 'TNFa' |
| 8 | 3230 | 'BCL6' | 'cjun' | 'CCL5' |
| 9 | 3230 | 'BCL6' | 'cjun' | 'IL5' |
| 10 | 3230 | 'CEBPB' | 'CCL5' | 'Nfkb1' |
| 11 | 3230 | 'CEBPB' | 'Nfkb1' | 'IL10' |
| 12 | 3230 | 'CEBPB' | 'Nfkb1' | 'il1b' |
| 13 | 3230 | 'IL5' | 'cjun' | 'stat1' |
| 14 | 3230 | 'IL10' | 'stat1' | 'CCL2' |
| 15 | 3230 | 'cjun' | 'TNFa' | 'stat1' |
| 16 | 3230 | 'cjun' | 'stat1' | 'CCL5' |
| 17 | 3230 | 'cjun' | 'stat1' | 'CCL2' |
| 18 | 200200 | 'CEBPB' | 'CCL5' | 'cjun' |
| 19 | 3003330 | 'BCL6' | 'cjun' | 'stat1' |
| 20 | 3003330 | 'CEBPB' | 'Nfkb1' | 'nfkb2' |
| 21 | 3003330 | 'CCR7' | 'CXCL12' | 'CCL5' |
| 22 | 3003330 | 'CCR7' | 'CXCL12' | 'CCL2' |
| 23 | 3003330 | 'CCR7' | 'CXCL12' | 'CXCL10' |
| 24 | 3003330 | 'CXCL1' | 'CCL2' | 'CXCL10' |
| 25 | 3003330 | 'CXCL1' | 'CCL2' | 'IL8' |
| 26 | 3003330 | 'CXCL1' | 'CCL2' | 'CXCL12' |
| 27 | 3003330 | 'CXCL1' | 'CCL2' | 'CCL20' |
| 28 | 3003330 | 'CXCL1' | 'CCL2' | 'CCL7' |
| 29 | 3003330 | 'CXCL1' | 'CCL2' | 'IL5' |
| 30 | 3003330 | 'CXCL1' | 'CCL2' | 'CXCL2' |
| 31 | 3003330 | 'CXCL1' | 'CXCL10' | 'CXCL12' |
| 32 | 3003330 | 'CXCL1' | 'IL8' | 'CXCL5' |
| 33 | 3003330 | 'CXCL1' | 'IL8' | 'CCL7' |
| 34 | 3003330 | 'CXCL1' | 'IL8' | 'CCL4' |
| 35 | 3003330 | 'CXCL1' | 'IL8' | 'IL1RN' |
| 36 | 3003330 | 'CXCL1' | 'IL8' | 'CXCL3' |
| 37 | 3003330 | 'CXCL1' | 'IL8' | 'CXCL2' |
| 38 | 3003330 | 'CXCL2' | 'CCL2' | 'IL8' |
| 39 | 3003330 | 'CXCL2' | 'CCL2' | 'CXCL12' |
| 40 | 3003330 | 'CXCL2' | 'CCL2' | 'CCL20' |
| 41 | 3003330 | 'CXCL2' | 'CCL2' | 'CCL7' |
| 42 | 3003330 | 'CXCL2' | 'CCL2' | 'IL5' |
| 43 | 3003330 | 'CXCL2' | 'IL8' | 'CXCL5' |
| 44 | 3003330 | 'CXCL2' | 'IL8' | 'CCL7' |
| 45 | 3003330 | 'CXCL2' | 'IL8' | 'CCL4' |
| 46 | 3003330 | 'CXCL2' | 'IL8' | 'CXCL10' |
| 47 | 3003330 | 'CXCL2' | 'IL8' | 'IL1RN' |
| 48 | 3003330 | 'CXCL2' | 'IL8' | 'CXCL3' |
| 49 | 3003330 | 'CXCL3' | 'IL8' | 'CXCL5' |
| 50 | 3003330 | 'CXCL3' | 'IL8' | 'CCL7' |
| 51 | 3003330 | 'CXCL3' | 'IL8' | 'CCL4' |
| 52 | 3003330 | 'CXCL3' | 'IL8' | 'CXCL10' |
| 53 | 3003330 | 'CXCL3' | 'IL8' | 'IL1RN' |
| 54 | 3003330 | 'IL1A' | 'LTA' | 'IL1R1' |
| 55 | 3003330 | 'IL1A' | 'LTA' | 'TNFa' |
| 56 | 3003330 | 'IL1A' | 'LTA' | 'LTB' |
| 57 | 3003330 | 'IL1A' | 'LTA' | 'IL10' |
| 58 | 3003330 | 'IL1A' | 'LTA' | 'il1b' |
| 59 | 3003330 | 'IL1A' | 'IL1R1' | 'IL1RN' |
| 60 | 3003330 | 'IL1A' | 'IL1R1' | 'il1b' |
| 61 | 3003330 | 'il1b' | 'LTA' | 'IL1R1' |
| 62 | 3003330 | 'il1b' | 'LTA' | 'TNFa' |
| 63 | 3003330 | 'il1b' | 'LTA' | 'LTB' |
| 64 | 3003330 | 'il1b' | 'LTA' | 'IL10' |
| 65 | 3003330 | 'il1b' | 'IL1R1' | 'IL1RN' |
| 66 | 3003330 | 'IL1R1' | 'IL1RN' | 'IL10' |
| 67 | 3003330 | 'IL1R1' | 'IL1RN' | 'IL8' |
| 68 | 3003330 | 'IL1RN' | 'IL10' | 'IL8' |
| 69 | 3003330 | 'IL1RN' | 'IL10' | 'stat1' |
| 70 | 3003330 | 'IL1RN' | 'IL10' | 'LTA' |
| 71 | 3003330 | 'IL1RN' | 'IL8' | 'CXCL5' |
| 72 | 3003330 | 'IL1RN' | 'IL8' | 'CCL7' |
| 73 | 3003330 | 'IL1RN' | 'IL8' | 'CCL4' |
| 74 | 3003330 | 'IL1RN' | 'IL8' | 'CXCL10' |
| 75 | 3003330 | 'IL5' | 'CCL2' | 'IL13' |
| 76 | 3003330 | 'IL5' | 'CCL2' | 'CXCL12' |
| 77 | 3003330 | 'IL5' | 'CCL2' | 'CCL20' |
| 78 | 3003330 | 'IL5' | 'CCL2' | 'CCL7' |
| 79 | 3003330 | 'IL5' | 'IL13' | 'IL13RA1' |
| 80 | 3003330 | 'IL8' | 'CXCL5' | 'CCL7' |
| 81 | 3003330 | 'IL8' | 'CXCL5' | 'CCL4' |
| 82 | 3003330 | 'IL8' | 'CXCL5' | 'CXCL10' |
| 83 | 3003330 | 'IL8' | 'CCL7' | 'CCL4' |
| 84 | 3003330 | 'IL8' | 'CCL7' | 'CXCL10' |
| 85 | 3003330 | 'IL8' | 'CCL7' | 'CCL8' |
| 86 | 3003330 | 'IL8' | 'CCL7' | 'CCL2' |
| 87 | 3003330 | 'IL8' | 'CCL4' | 'CXCL10' |
| 88 | 3003330 | 'IL8' | 'CXCL10' | 'CXCL12' |
| 89 | 3003330 | 'IL10' | 'stat1' | 'LTA' |
| 90 | 3003330 | 'IL10' | 'stat1' | 'cjun' |
| 91 | 3003330 | 'IL10' | 'LTA' | 'TNFa' |
| 92 | 3003330 | 'IL10' | 'LTA' | 'LTB' |
| 93 | 3003330 | 'CXCL10' | 'CXCL12' | 'CCL5' |
| 94 | 3003330 | 'CXCL10' | 'CXCL12' | 'CCL2' |
| 95 | 3003330 | 'CCL2' | 'CXCL12' | 'CCL20' |
| 96 | 3003330 | 'CCL2' | 'CXCL12' | 'CCL7' |
| 97 | 3003330 | 'CCL2' | 'CXCL12' | 'CCL5' |
| 98 | 3003330 | 'CCL2' | 'CCL20' | 'CCL7' |
| 99 | 3003330 | 'CCL2' | 'CCL7' | 'CCL8' |
| 100 | 30300200 | 'CEBPB' | 'CCL5' | 'CXCL12' |
| 101 | 30300200 | 'CEBPB' | 'IL10' | 'stat1' |
| 102 | 30300200 | 'CEBPB' | 'IL10' | 'LTA' |
| 103 | 30300200 | 'CEBPB' | 'IL10' | 'IL1RN' |
| 104 | 30300200 | 'CEBPB' | 'il1b' | 'LTA' |
| 105 | 30300200 | 'CEBPB' | 'il1b' | 'IL1R1' |
| 106 | 30300200 | 'CXCL1' | 'CCL2' | 'stat1' |
| 107 | 30300200 | 'CXCL2' | 'CCL2' | 'stat1' |
| 108 | 30300200 | 'IL5' | 'CCL2' | 'cjun' |
| 109 | 30300200 | 'IL5' | 'CCL2' | 'stat1' |
| 110 | 30300200 | 'IL5' | 'cjun' | 'IL13' |
| 111 | 30300200 | 'cjun' | 'TNFa' | 'LTB' |
| 112 | 30300200 | 'cjun' | 'TNFa' | 'LTA' |
| 113 | 30300200 | 'cjun' | 'CCL5' | 'CXCL12' |
| 114 | 30300200 | 'CCL2' | 'stat1' | 'CXCL12' |
| 115 | 30300200 | 'CCL2' | 'stat1' | 'CCL20' |
| 116 | 30300200 | 'CCL2' | 'stat1' | 'CCL7' |
| 117 | 33303330 | 'CXCL1' | 'CXCL10' | 'IL8' |
| 118 | 33303330 | 'LTA' | 'TNFa' | 'LTB' |
