# Supplementary material for: Induction of an Inflammatory Loop by Interleukin-1β and Tumor Necrosis Factor-α Involves NF-kB and STAT-1 in Differentiated Human Neuroprogenitor Cells
Source: PLoS One. 2013 Jul 29;8(7):e69585. doi: 10.1371/journal.pone.0069585 (PMC3726669; doi:10.1371/journal.pone.0069585)
Supplement: Table S3 — Network motifs (NMs) with a transcription factor. Identified 40 NMs with more than one transcription factor are listed. (DOCX) [file pone.0069585.s003.docx]

**Supplementary table S3: Network motifs (NMs) with a transcription factor:** Identified NMs with more than one transcription factor are listed.

|  |  | | | | | | | |  |  | |  |
| --- | --- | --- | --- | --- | --- | --- | --- | --- | --- | --- | --- | --- |
|  | **Node 1** | **Node 2** | **Node 3** |  | |  | | **Node 1** | **Node 2** | **Node 3** |  | |
| 1 | 'CEBPB' | 'CCL5' | 'IL10' |  | | 21 | | 'IL1RN' | 'IL10' | 'stat1' |  | |
| 2 | 'CEBPB' | 'CCL5' | 'il1b' |  | | 22 | | 'IL10' | 'stat1' | 'LTA' | * | |
| 3 | 'CEBPB' | 'IL10' | 'il1b' |  | | 23 | | 'IL10' | 'stat1' | 'cjun' |  | |
| 4 | 'IL5' | 'cjun' | 'TNFa' |  | | 24 | | 'CEBPB' | 'CCL5' | 'CXCL12' | * | |
| 5 | 'IL5' | 'cjun' | 'CCL5' |  | | 25 | | 'CEBPB' | 'IL10' | 'stat1' | |  |
| 6 | 'cjun' | 'TNFa' | 'CCL5' |  | | 26 | | 'CEBPB' | 'IL10' | 'LTA' | |  |
| 7 | 'BCL6' | 'cjun' | 'TNFa' | * | | 27 | | 'CEBPB' | 'IL10' | 'IL1RN' | |  |
| 8 | 'BCL6' | 'cjun' | 'CCL5' | * | | 28 | | 'CEBPB' | 'il1b' | 'LTA' | |  |
| 9 | 'BCL6' | 'cjun' | 'IL5' | * | | 29 | | 'CEBPB' | 'il1b' | 'IL1R1' | |  |
| 10 | 'CEBPB' | 'CCL5' | 'Nfkb1' | * | | 30 | | 'CXCL1' | 'CCL2' | 'stat1' | |  |
| 11 | 'CEBPB' | 'Nfkb1' | 'IL10' | * | | 31 | | 'CXCL2' | 'CCL2' | 'stat1' | |  |
| 12 | 'CEBPB' | 'Nfkb1' | 'il1b' | * | | 32 | | 'IL5' | 'CCL2' | 'cjun' | |  |
| 13 | 'IL5' | 'cjun' | 'stat1' | * | | 33 | | 'IL5' | 'CCL2' | 'stat1' | |  |
| 14 | 'IL10' | 'stat1' | 'CCL2' |  | | 34 | | 'IL5' | 'cjun' | 'IL13' | |  |
| 15 | 'cjun' | 'TNFa' | 'stat1' | * | | 35 | | 'cjun' | 'TNFa' | 'LTB' | |  |
| 16 | 'cjun' | 'stat1' | 'CCL5' | * | | 36 | | 'cjun' | 'TNFa' | 'LTA' | |  |
| 17 | 'cjun' | 'stat1' | 'CCL2' | * | | 37 | | 'cjun' | 'CCL5' | 'CXCL12' | |  |
| 18 | 'CEBPB' | 'CCL5' | 'cjun' | * | | 38 | | 'CCL2' | 'stat1' | 'CXCL12' | |  |
| 19 | 'BCL6' | 'cjun' | 'stat1' | * | | 39 | | 'CCL2' | 'stat1' | 'CCL20' | |  |
| 20 | 'CEBPB' | 'Nfkb1' | 'nfkb2' | * | | 40 | | 'CCL2' | 'stat1' | 'CCL7' | |  |
|  |  |  |  | |  |  |  | |  |  | |  |
|  | * | NMs with more than one TFs | | | | |  | |  |  | |  |
